# Vulvo-Vaginitis in Infants and Children

**Published:** 1913-09

**Authors:** W. N. Adkins

**Affiliations:** Atlanta, Ga.


### VULVO-VAGINITIS IN INFANTS AND CHILDREN.*

By W. N. Adkins, M. D., Atlanta, Ga.

In this paper I speak of only two types of vulvo-vaginitis in infants and children: the specific or gonorrheal, and the non-specific. The latter type is usually a simple catarrhal condition of the membranous lining of the vaginal tract, sometimes including the cervical canal and uterus. This condition is usually found in children in an anaemic and debilitated condition. It is sometimes caused primarily by irritation from the migration of seat worms.

Specific vulvo-vaginitis often follows the acute infectious diseases, particularly measles and scarlet fever. Both these diseases are of the desquamative type, and this appears to have considerable influence in preparing a fertile ground, as the internal organs desquamate somewhat. It therefore follows that children recovering from these diseases are more prone to infection of the gonococcus, and also during the acute stages, on account of the inflammatory condition of the membranous linings. Most of my observations with vulvo-vaginitis have been with cases complicating or following measles, scarlet fever and diphtheria. I have treated and gathered statistics on about three hundred, but unfortunately lost the records, hence the statements made in this paper are from memory. The extreme contagiousness of specific vulvo-vaginitis, and the rapidity with which it spreads in hospital wards, is astounding. The infection is most certainly carried by the hands, clothing, thermometers, or by using the same toilets, bath tubs, or other such utensils that have not been properly cleansed. Once this infection starts in a ward, it spreads with alarming rapidity, regardless of all measures taken to prevent it. I remember in one of the wards at Willard Parker Hospital, at a time when the census was very heavy, this condition broke out and spread very rapidly, and before it could be checked, two boys of about three years of age contracted urethral infections. For obvious anatomical reasons, girls are more prone to the infection than are boys, but I can easily understand how boys could be infected by careless exposure. The two cases just mentioned are the only ones I have seen, and they occurred in spite of the fact that the cases were segregated in another ward, as fast as they were discovered. At this time the scarlet fever building was overcrowded, having about five hundred and fifty cases, when it was built to hold only four hundred. This severe outbreak was the cause of a rule being made that the nurses wear rubber gloves at all times when handling a case, and each patient had a separate thermometer, hung in a container over the crib or bed. Bowls of antiseptic solution were distributed at various points around the wards, for rinsing the hands. These rules proved very valuable later. They did not stop outbreaks from occurring, but a smaller percentage of infections followed.

All females admitted to a hospital, and particularly infants and children, should have careful vaginal smears made and examined by a competent bacteriologist, before being admitted into the wards to be a possible menace to the other patients; and if any vaginal discharge is present, the case should be isolated until the clinical signs cease and several smears are made and found repeatedly negative. The extreme importance of this routine was impressed upon me by the fact that careful investigations of all outbreaks, showed that the primary infection was from some newly admitted case, which showed no clinical signs at the time of admission. The discharge would appear within about three days, and upon interviewing some member of the family, the information was brought forth that the child previously had a vaginal discharge, which had ceased before she was sent to the hospital. Sometimes we could get the father to admit that he had a urethral discharge. All this goes to prove that the fact that the absence of visible discharge does not necessarily mean that there is no infection present. Such examples will accentuate the value of the method of taking smears, that I am about to relate.

The late Dr. Ira VanGeison of New York, who was connected with the Health Department Laboratories during my service in the hospitals, devised a method of taking vaginal smears from infants and children which is absolutely reliable.

Use a small glass pipette, with a rubber bulb attached like a medicine dropper, insert the small end into the vagina, inject a small amount of weak bichloride solution into the vagina, and back into the tube several times, until the solution is thoroughly mixed with the contents of the vagina. Smear a small quantity on a glass slide and you will find it makes a smoother and more reliable smear than can be made with a swab. I have often seen suspected cases with negative smears show a typically positive smear when this method was resorted to. My experience with vaginal smears has taught me that the ordinary way of making a smear with a cotton swab is most unreliable, and if anatomical conditions warrant it, the material for the smear should be taken from the vagina and cervix.

Rubin and Leopold of New York, made extensive and practical investigations with fifty cases, to learn the cause of the persistence of gonorrheal vulvo-vaginitis in children, and their most excellent article on the subject appeared in the January issue of the American Journal of Diseses of Children. They were most thorough in their methods and kept all their cases under careful and constant scrutiny. They employed an electrically lighted female urethroscope of proper size, in order to make macroscopical examination of the vagina and cervix. They found contrary to the claims of some authorities, that the cervix was not immune to the attack. They also claim that the hymen does not form an effectual barrier to the infection, as is maintained by some authors, and they found it atresic in rare instances. They also found that infection of the cervix occurred in the majority of cases investigated by them. I have seen a few cases where the hymen appeared completely atresic and still there was a vaginal discharge, and upon careful scrutiny, found an opening behind a fold, from which pus was found issuing in quantity.

It seems that little can be done in the way of local or constitutional treatment, which will give any improvement permanently, and the disease seems to run its course in spite of all one may do to check it. In 1909, I put thirty-two cases through a thorough course with the vaccines, with no good results as far as could be noticed. I then used vaginal injections of the solution of lactic acid bacilli, with slight improvement. I then irrigated these cases twice daily with a weak permanganate of potash, followed with 10% argyrol, which was left in the vagina. I used a small soft rubber catheter for an irrigating tip, and had no difficulty in effecting an entrance in any case. The patients were all kept in bed. This latter treatment benefitted most of them temporarily, some apparently permanently; but when a few of the latter were seen after being out of the hospital about six months, I found them all to have had a recurrence. Some of the cases appeared to get well without treatment, and had negative smears, but the discharge appeared later and the smears again became positive. Some authorities claim the only benefit is derived by absolute rest in bed; I have never seen any case benefitted by any treatment that was not followed up. Others claim no irrigation should be used; that it not only does no good, but does harm, in that it serves to attract the attention of the child to its genital organs.

Text books speak of rheumatism and arthritis as complications; I have never seen either, but have seen quite a few eye infections, usually where the hands were not fastened. The eye complications, I should think, the most dreaded of all, and the one to be most guarded against.

It is an accepted fact that gonorrhoea in the adult female usually becomes chronic; then why should this not be the case in infants and children? The latter two on account of the more delicate structures of the vaginal tract, must certainly be more prone to the infection, and granting when once established the infection appears incurable, should we not use every possible precaution to protect these poor helpless innocents from accidental infection of so dreaded a disease?

We who live in the south are in more danger of having this difficulty to deal with owing to the fact that the majority of nurses of children are negro females. We all know that the majority of these people either have gonorrhea, usually in an infective state, or are exposing themselves almost daily, as virtue is an unknown quantity with them. And in addition to that deplorable state of affairs, which is a grave menace to a child who is daily in close contact with them, there is another most unpleasant feature; their lack of any appreciable effort at personal cleanliness and their utter disregard for the common laws of hygiene, due either to their total ignorance, or to pure carelessness and indifference. None of us here to-night would spend many moments in close proximity to a nurse exuding a most nauseating odor; then why should a helpless child who is unable to resist, be compelled to undergo such abhorrent experiences? I do not wonder that some of them vomit without any apparent cause. And this foul odor is not always due to lack of soap and water, but often to an existing vaginal discharge.

All physicians use prophylactic measures with an infant's eyes at birth; how many take this precaution with the vagina and vulva, even where gonorrhea is suspected in the mother or father? Any physician noticing a nurse with a bad cough or skin disease would certainly advise that her services be dispensed with, as a protection to the baby, but how many advise or insist upon having the negro nurse examined for venereal disease? Why should there not be a law compelling these nurses to undergo an examination by a physician, and hold certificates from him stating that they are free from venereal as well as other contagious and infectious diseases, before they be allowed to handle infants and children? I should think such a law would be most adequate and just, and it would better protect and safeguard the future wives and mothers. I firmly believe that if the laity were cognizant of the dangers their children were daily subjected to in this manner, such a law would not be long in forthcoming. It seems to me that it is the duty of the medical profession to enlighten them and to start the movement.

From my experience with gonorrheal vulvo-vaginitis in hospital practice, I have arrived at the following conclusions:

That the disease is highly infectious, and once started, spreads rapidly.

That any and all forms of treatment so far applied, seem to be of little permanent benefit.

That all precautions should be taken to protect infants and children from accidental infection, and that no nurse be allowed to handle them, who has venereal disease.

That the vulva and vagina are probably even more prone to the infection than the eyes, and that the same precautions should be used for both.

618 Forsyth Building.

---

The absence for a long period of time, of all signs of local infection in a case of purulent prostatitis, is strongly suggestive of a calculous etiology.

